# Supplementary material for: Effect of Proofing on the Rheology and Moisture Distribution of Corn Starch-Hydroxypropylmethylcellulose Gluten-Free Dough
Source: Foods. 2023 Feb 6;12(4):695. doi: 10.3390/foods12040695 (PMC9956097; doi:10.3390/foods12040695)

Supplementary data

Supplementary Table S1 Effect of proofing on the crystallinity value of CS–HPMC gluten-free dough.

Sample	D-0	D-30	D-60	D-90
**CV**	28.09±0.59a	27.63±1.05a	27.73±0.91a	24.55±0.23b

Data followed by the same letter in the same column are not significantly different ($p > 0.05$). CV: crystallinity value.

Supplementary Figure S1 The HPLC chromatogram of the CS–HPMC gluten-free dough after proofing for 30 min. Peak 1, arabinose; peak 2, glucose; peak 3, fructose; peak 5, mannose.

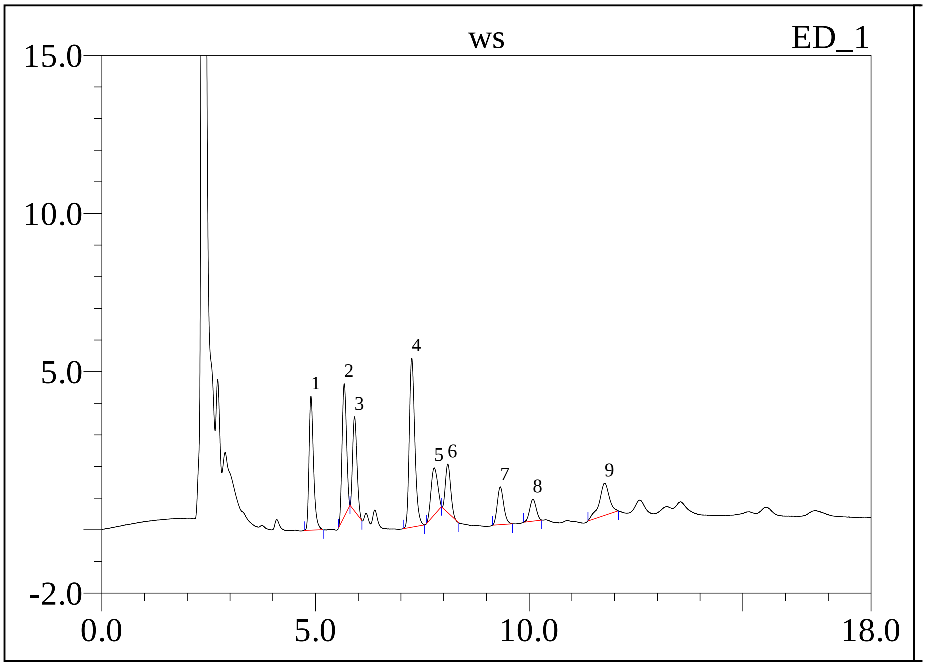

Supplementary Figure S2 Effect of proofing on the creep-recovery curves of CS–HPMC gluten-free doughs.

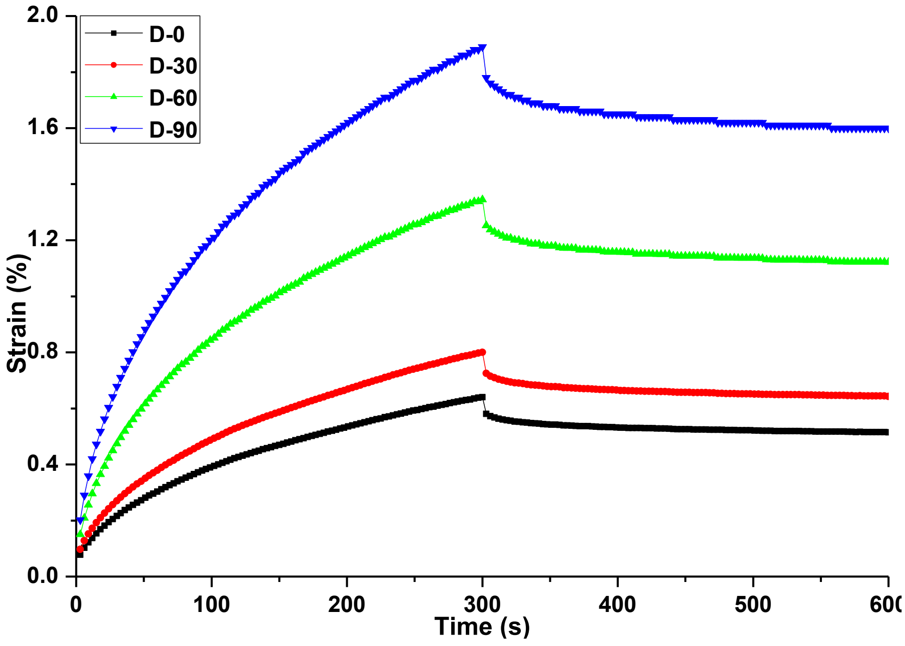

Supplement: Supplementary file 1 [file foods-12-00695-s001.zip › foods-2117718-supplementary.pdf]
